# Artificial Intelligence and Its Role in Predicting Periprosthetic Joint Infections

**DOI:** 10.3390/biomedicines13081855

**Published:** 2025-07-30

**Authors:** Diana Elena Vulpe, Catalin Anghel, Cristian Scheau, Serban Dragosloveanu, Oana Săndulescu

**Affiliations:** 1 The “Carol Davila” University of Medicine and Pharmacy, 050474 Bucharest, Romania; diana-elena.plescan@drd.umfcd.ro (D.E.V.); oana.sandulescu@umfcd.ro (O.S.); 2 Department of Orthopaedics, “Foisor” Clinical Hospital of Orthopaedics, Traumatology and Osteoarticular TB, 021382 Bucharest, Romania; 3 Department of Computer Science and Information Technology, “Dunarea de Jos” University of Galati, 800146 Galati, Romania; catalin.anghel@ugal.ro; 4 Department of Radiology and Medical Imaging, “Foisor” Clinical Hospital of Orthopaedics, Traumatology and Osteoarticular TB, 021382 Bucharest, Romania; 5 National Institute for Infectious Diseases “Prof. Dr. Matei Bals”, 021105 Bucharest, Romania; 6 Academy of Romanian Scientists, 050044 Bucharest, Romania

**Keywords:** periprosthetic joint infection, artificial intelligence, machine learning, arthroplasty, joint

## Abstract

Periprosthetic joint infections (PJIs) represent one of the most problematic complications following total joint replacement, with a significant impact on the patient’s quality of life and healthcare costs. The early and accurate diagnosis of a PJI remains the key factor in the management of such cases. However, with traditional diagnostic measures and risk assessment tools, the early identification of a PJI may not always be adequate. Artificial intelligence (AI) algorithms have been integrated in most technological domains, with recent integration into healthcare, providing promising applications due to their capability of analyzing vast and complex datasets. With the development and implementation of AI algorithms, the assessment of risk factors and the prediction of certain complications have become more efficient. This review aims to not only provide an overview of the current use of AI in predicting PJIs, the exploration of the types of algorithms used, and the performance metrics reported, but also the limitations and challenges that come with implementing such tools in clinical practice.

## 1. Introduction

Total joint replacement (TJR) surgeries have become one of the most frequently performed surgeries in the orthopedic field over the years. Because of the high success rate, high patient satisfaction, and rapid recovery, the volume of primary TJRs is on a continuous rise. A recent article studying projections and epidemiology of primary arthroplasty stated that the number of annual total hip arthroplasties (THAs) and total knee arthroplasties (TKAs) has risen by 177% and 156%, respectively, between 2000 and 2019 [1]. Given these numbers, complications associated with these procedures have also seen a rise in prevalence, with periprosthetic joint infections (PJIs) being one of the most common and problematic complications, both for the patient and the health system. The incidence of PJIs is stated to fall between 1% and 3% in the United States, but it often leads to catastrophic outcomes [2].

Infections are responsible for 10.9% to 13.7% of revision surgeries after THAs, with slightly higher economic implications for TKAs, associated with serious physical and psychological impairment, but also huge economic costs affecting the patient’s family and the healthcare system [3]. It is estimated that the total cost of a septic revision surgery is double that of an aseptic one [4]. These high costs have a huge impact on the healthcare system, with a longer length of stay in the hospital for revision procedures and higher medical and surgical costs. The revision burden due to PJIs underwent an important rise in the USA, but also in Europe and Korea, from an annual total cost of USD 372.7 million in 2002 to USD 902.9 million in 2017 in the USA [5,6,7]. Table 1 provides an insight into the actual costs associated with revision TJA due to PJIs. Early detection, accurate diagnosis, and effective treatment can provide high success rates. However, as the clinical presentation of a PJI can be variable, from acute inflammatory to chronic disease, and adding the lack of a generally accepted definition of PJI makes the accurate and early diagnosis extremely difficult. Major scientific societies, such as The Musculo-Skeletal Infections Society, The International Consensus Meeting, and The European Bone and Joint Infection Society, have each released their own PJI definition, with slightly different diagnostic criteria, making the challenge of an early and accurate infectious diagnosis even more difficult [8]. The rise of artificial intelligence (AI) and machine learning models, and their use in medical diagnosis and treatment, can become useful tools in the diagnosis and treatment of PJIs.

**Figure 1:**
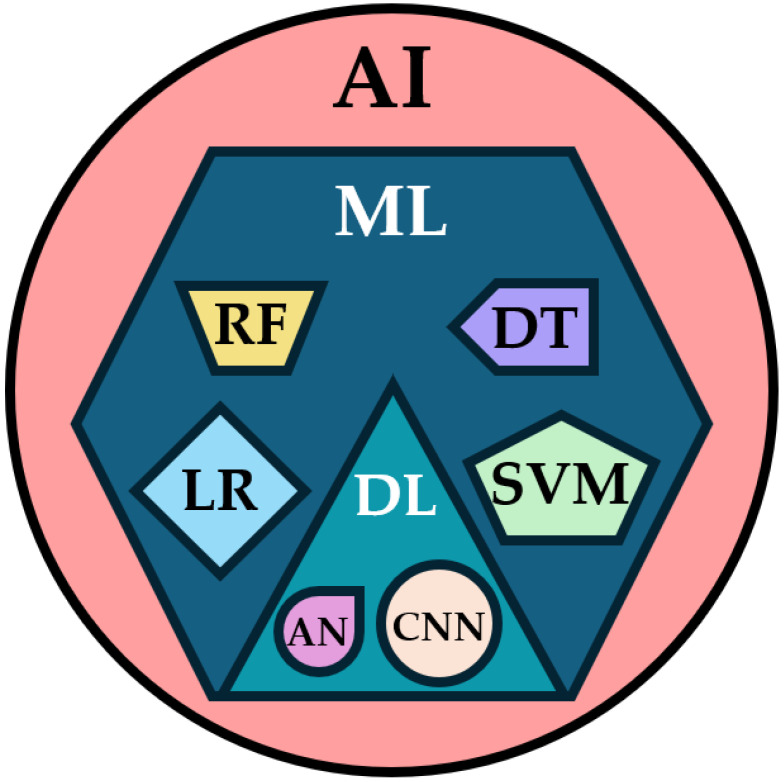
The schematic diagram representing the relationship between artificial intelligence (AI), machine learning (ML), and deep learning (DL). AN = artificial network; CNN = convoluted neural network; DT = decision tree; LR = logistic regression; RF = random forest; SVM = support vector machine.

The use of AI has risen in technological domains, especially aviation and other industries, and has gained significance in healthcare as well [9,10,11]. AI represents a big umbrella term, that constitutes of subfields, including machine learning (ML), which further contains a subfield that is labeled as deep learning (DL) (Figure 1) [12]. ML is a part of AI based on pattern recognition and learning theory [13]. ML delineates algorithms that can make projections and acknowledge correlations by identifying non-linear relationships in big datasets [14]. Various machine learning algorithms have been assessed in different medical domains, but particularly in image analysis, and ML has been suggested to surpass medical specialists, when carefully curated [15].

In orthopedics, the use of AI and ML has been initiated, with a rising number of published studies in this field, which highlight the potential of these technologies for becoming important tools in the clinical and surgical life of orthopedic specialists. The integration of AI in orthopedic surgery and the management of PJIs has shown promise in several key areas, including early detection of infection, development of risk predictive models for PJIs, intraoperative surveillance through sensor technology and data integration, postoperative monitoring, and personalized treatment strategies.

Although several review articles have examined the use of artificial intelligence in orthopedic surgery and infectious disease diagnosis, only a limited number have focused specifically on periprosthetic joint infections. Previous reviews have discussed AI applications in joint arthroplasty broadly or emphasized specific aspects such as radiological interpretation or the use of robotics and advanced technologies in total hip or knee arthroplasties [16,17,18]. However, these reviews lack an integrated, comparative analysis of various AI and machine learning (ML) approaches across multiple types of clinical data (e.g., imaging, serum biomarkers, electronic health records data, etc.), and do not evaluate their diagnostic performance in the context of the difficulties and challenges posed by PJI diagnostic.

This narrative review focuses on the analysis of different artificial intelligence tools, machine learning models, and their potential use in predicting PJIs, and aims to evaluate the current landscape of artificial intelligence and machine learning applications in the diagnosis and management of periprosthetic joint infections. In this article, we review the implementation of different AI techniques for the early and accurate diagnosis of periprosthetic joint infections (PJIs). The literature regarding these new and exciting tools is rising, so the need for an integrative, comparative analysis of AI approaches across different types of clinical data is evident. By providing this comprehensive analysis, our review aims to serve as a practical resource for clinicians and healthcare administrators interested in the potential of AI to improve outcomes in patients at risk of a periprosthetic joint infection.

### Search Strategy and Selection Criteria of the Articles Included

**Figure 2:**
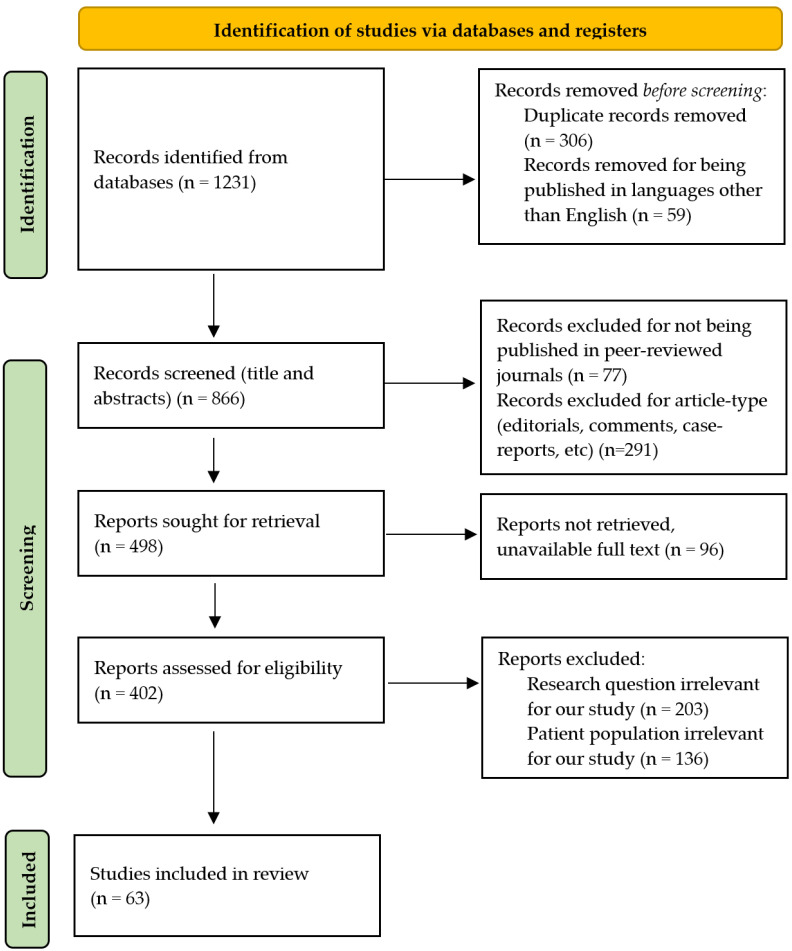
PRISMA flow diagram of the selected studies.

The databases PubMed, Google Scholar, and Scopus were searched using a combination of the following keywords to identify the relevant literature: “periprosthetic joint infection”, “replacement”, “arthroplasty”, “infection”, “artificial intelligence”, “machine learning”, “PJI”, “THA infection”, and “TKA infection”. The inclusion timeframe was considered between January 2014 and May 2025. To identify relevant articles, an initial screening was performed by analysis of titles and abstracts. The full-text articles of the selected eligible pool were then evaluated. The criteria used for excluding the articles were as follows: non-English publications, unavailability of full texts, articles with no clear information or lacking data, editorials, comments, and non-clinical research (Figure 2).

## 2. Machine Learning Models Used for the Early Detection of PJIs Using Imagistic Data

ML algorithms can perform predictions based on a large dataset and can learn patterns without being programmed to perform in this manner. The use of ML regarding PJIs proves helpful by its capability to analyze vast amounts of data, in order to identify small changes and extremely early signs of infection, which could not be determined by traditional methods [19]. In this way, the diagnosis is achieved faster and earlier, preventing the progression of an infection and leading to better patient outcomes.

**Figure 3:**
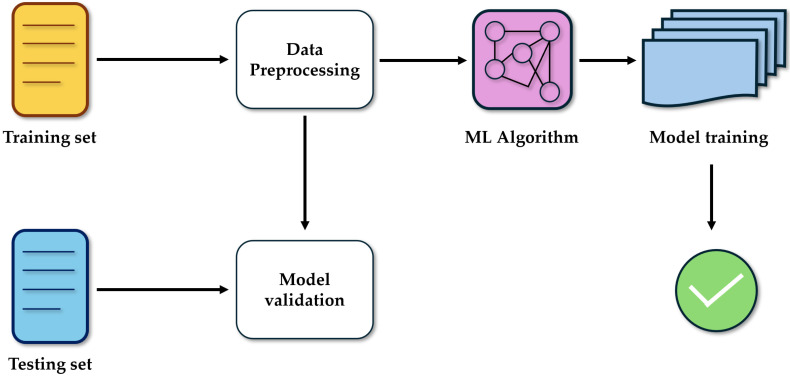
The illustration of the process of how machine learning models use data for training and testing.

ML algorithms possess the capacity to learn from examples by regulating and improving associations in order to enhance their accuracy. The input data that are used to build the ML algorithm are usually divided into several datasets. The learning phase is conducted on a subset of data, denominated the “training set”, which is represented by a set of examples that have the role of fitting the parameters of the model. Then, the ML algorithm is used on a “validation dataset”, in order to finely tune the parameters of the model. And, finally, the algorithm is tested on the “testing set”, on which the performance is evaluated, in order to provide an unbiased assessment of the final algorithm. This training process can be either supervised or unsupervised. In a supervised training process, the data used are characterized and tagged by humans. In the unsupervised training process, the data used are not labeled and unknown, but the result is acknowledged (Figure 3) [12,20].

Imaging diagnosis plays an important role in the early detection of PJIs. Previous studies have already demonstrated the importance of computer tomography (CT) or magnetic resonance imaging (MRI) in PJI diagnosis [21,22]. In addition, several newer advanced nuclear medicine approaches, such as bone scintigraphy (BS), leukocyte scintigraphy (LS), anti-granulocyte antibody imaging (AGS), and fluorodeoxyglucose positron emission tomography (FDG-PET/CT), can help with the diagnosis of PJIs [23].

Before presenting how specific machine learning algorithms are applied to imaging data, it is important to briefly outline some commonly used AI methods in medical diagnostics. Logistic regression (LR) is a basic classification technique that estimates the probability of infection based on input features, such as lab values or clinical risk factors [24]. Random forest (RF) is an ensemble method using multiple decision trees to improve prediction accuracy and robustness [25]. A support vector machine (SVM) is a powerful algorithm that separates infected from non-infected cases by finding an optimal hyperplane in a high-dimensional space; it is particularly effective for structured datasets and has been used in MRI analysis [26]. Finally, deep learning (DL) methods, including artificial neural networks and convolutional neural networks (CNNs), are particularly suitable for image data or unstructured clinical notes, offering high performance in complex pattern recognition tasks [27].

### 2.1. Radiographic Images

The radiographic examination is the first classical line of diagnosis after total arthroplasties, and the first and foremost imaging examination for postoperative complications, but with questionable utility. Relevant radiographic findings in cases with PJIs are the signs of implant loosening, radiolucency at the implant–bone interface, reaction of the periosteum, and osteolysis [28]. However, most radiographic modifications become visible after a minimum of 30% bone loss, with most early PJIs having a normal radiographic examination [29]. In cases of high radiographic suspicion of infection, the next step to confirm the diagnosis is typically an image-guided aspiration from the site [30]. However, manual interpretation of images and radiographs may not always divulge subtle signs of infection, with a high interindividual subjectivity regarding the interpretation of the images. In order to minimize this problem and to provide an accurate and early diagnosis, several machine learning models have been used that can enhance the analysis of imaging data. Within the ML models, DL algorithms have shown promising results in analyzing imaging examinations and gained recognition within the radiologic imaging community.

**Figure 4:**
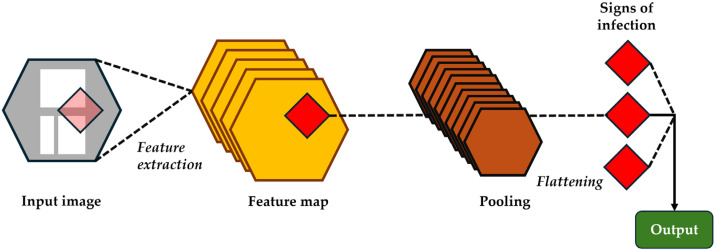
Graphical representation of how convolutional neural networks work and identify patterns within imaging data.

Convolutional neural networks (CNNs), also known as ConvNets, represent a type of DL that uses data for image recognition and classification. Figure 4 presents a simplified explanation of the way CNNs work in order to identify patterns within an image.

CNNs can be used for the analysis of musculoskeletal radiographic images to automatically detect specific signs of joint infection [31]. These can detect early signs of PJIs by recognizing patterns in the images analyzed after training on a vast amount of medical imaging. CNNs can identify different patterns, such as the presence of radiolucency, implant loosening, periosteal reaction, or osteolysis [32]. In a study using a CNN built to detect loosening based on anteroposterior and lateral view radiographs of THAs and TKAs achieved an accuracy, sensitivity, and specificity of 88.3%, 70.2%, and 95.6%, respectively [33].

### 2.2. Computer Tomography Imaging

Computer tomography (CT) plays an important role as a diagnostic tool for PJIs, by providing superior contrast between soft tissue and bones, offering improved recognition of periarticular soft tissue and bone anomalies, compared to the radiographic examination [34]. The role of CT imaging in the early detection of PJIs is the accurate detection of soft tissue abscesses, joint effusions, fluid accumulation in periarticular muscle and fat tissue, and identification of the difference between septic and aseptic implant loosening; however, metal artifacts can decrease the image quality and increase the radiation exposure during examination, and these are important limitations of the method [35,36]. CT is also the most used imaging technique for guided bone biopsies, facilitating an accurate diagnosis [37]. Li et al. have developed a fusion method, named Hierarchical GCN-Based Transformer, that is based on DL and multimodal techniques, which has the ability to integrate text data and CT images, and facilitates the rising interest in the capability to diagnose PJIs [38]. Studies using CT and the use of AI technology to integrate the imaging findings with the diagnosis of infection are still insufficient, and additional research is required.

### 2.3. MRI Imaging

Magnetic resonance imaging (MRI) provides an outstanding contrast of the bone–implant interface and soft tissue envelope. MRI is used to detect edema of the bone, capsule, and muscles, the periosteal response, joint effusion, and soft tissue collection [39,40]. Galley et al. have evidenced a sensitivity of 78% to 95% and a specificity of 73% to 95%, by using MRI with metal artifact reduction for the imaging diagnosis of PJIs, producing high-resolution images that provide detailed soft tissue evaluation [39]. Moreover, studies using experimental rabbit models have shown that the MRI investigation can reveal evidence of PJI as early as one week after deliberately infecting the joint [41]. The role of MRI imaging in predicting infection following a total hip arthroplasty has been established in a study on 173 patients carried out by Albano et al. An ML algorithm based on a support vector machine (SVM) was used and trained on a cohort of 117 patients, achieving 92% sensitivity and 62% specificity, and then tested on the testing cohort of 53 patients, achieving 92% sensitivity and 79% specificity [42].

### 2.4. Advanced Nuclear Medicine Techniques

Bone scintigraphy (BS) is a diagnostic tool that uses nuclear tracers (usually technetium-99m labeled with phosphate), which can gather in areas with high blood flow and bone remodeling processes, and therefore detects infection and bone metastases with a high sensitivity [43]. A three-phase bone scintigraphy (TPBS) is performed to asses early perfusion, diffusion (or blood pool), and late bone uptake. According to the latest consensus document gathered by the European Association of Nuclear Medicine (ENAM), European Bone and Joint Infection Society (EBJIS), and the European Society of Radiology (ESR), a negative TPBS rules out a PJI [29]. However, TPBS has a high sensitivity but a low specificity for PJIs, as a positive result can also be obtained in a wide range of pathologies. Studies using TPBS for the diagnosis of PJIs in THAs and TKAs reported a sensitivity of 81% and 75%, respectively, and a specificity of 78% and 55%, respectively [44,45]. Bone remodeling after a total arthroplasty is present for at least one year after implantation, and so a false-positive BS may be present due to normal bone remodeling in the first postoperative year [46]. In a study of 449 patients with THAs and TKAs, Nie et al. developed a PJI diagnostic method based on Dynamic Bone Scintigraphy and Effective Neural Network (DBS-eNET), and compared it with CNNs and three nuclear medicine physicians. The DBS-eNET achieved higher diagnostic scores than the CNNs, comparable to those of the nuclear medicine physicians, suggesting potential clinical value for use in the future [47].

White blood cell scintigraphy (WBCS) or leukocyte scintigraphy (LS) is a type of nuclear medicine exploration that uses labeled WBCs, and acquires images at three different time points (early: 30–60 min; delayed: 2–4 h; and late: 20–24 h) [8]. This technique can achieve a high sensitivity and a specificity in the diagnosis of PJIs by using several image acquisition parameters and interpretation criteria [48]. In a recent meta-analysis, LS achieved a sensitivity of 88% and a specificity of 77% in the correct assessment of PJIs in total knee arthroplasties [45]. Table 2 contains a comparative result of the most important articles that studied different AI tools and applications in a clinical setting, and their performance achieved in the early detection of PJIs.

Anti-granulocyte antibody scintigraphy uses labeled monoclonal antibodies that target leukocyte antigens, and therefore accumulate in areas of inflammation. Only a few studies have used this specific type of scintigraphy on small cohorts of patients; therefore, further studies are necessary.

18F-fluorodeoxyglucose positron emission tomography (FDG/PET) measures the metabolic activity in different tissues, making it possible to identify musculoskeletal infections and PJIs. Although extremely useful in oncologic pathologies, FDG/PET can identify synovitis, loosening, and PJIs [49]. In a meta-analysis that comprised 1437 patients and 23 studies, FDG/PET/CT provided an important role in the diagnosis of PJIs, with a sensitivity of 85%, a specificity of 86%, and an accuracy of 92% [50].

Although both anti-granulocyte antibody scintigraphy and 18F-fluorodeoxyglucose positron emission tomography can diagnose PJIs with high accuracy, sensitivity, and specificity, the lack of these techniques in the common current diagnosis panel of infection yields limited results. As most ML methods rely on big datasets, to our knowledge, there is no study that utilizes AI in predicting PJIs based on such diagnostic tests; therefore, further research on this topic is necessary.

## 3. Machine Learning Models Used for the Early Detection of PJIs Based on Serum Biomarker Analysis

Biomarker analysis, such as C-reactive protein (CRP) levels, white blood cell count (WBC), erythrocyte sedimentation rate (ESR), or fibrinogen (FIB), is frequently used to assess general systemic inflammation and may help with risk assessment in infection after a total joint replacement. Due to the low cost of serological analysis and wide availability, these biomarkers are often used as an initial paraclinical investigation for patients with a painful joint arthroplasty [51]. Because these biomarkers are non-specific and can be elevated in other medical conditions, other more specific inflammatory biomarkers have been studied, such as procalcitonin (PCT), the polymorphonuclear leukocyte percentage (PMN%), the neutrophil-to-lymphocytes ratio (NLR), the platelet count-to-mean platelet volume ratio (PC/mPV), D-dimers, interleukin-6 (IL-6), the albumin/globulin ratio, or IL-1β and TNF-α serum levels [52].

Starting from the premise that serum biomarkers are easy to collect periodically, as limited sensitivity and specificity are reported in the current literature, Klim et al. have used a multi-biomarker model that applied logistic regression with lasso-regularization on 124 surgical procedures, using fibrinogen, C-reactive protein, the ratio of fibrinogen to CRP, and the ratio of thrombocytes to CRP. Results showed no increase in diagnostic accuracy by combining biomarkers such as C-reactive protein and fibrinogen as single markers [53].

In another paper, in a total of 133 included cases, 18 preoperative blood biochemical tests were used to differentiate between PJIs and non-PJIs. A random forest classifier was used to discriminate PJIs and non-PJIs, with C-reactive protein, total protein, and blood urea nitrogen being the most important features to separate the two groups [54].

Another ML algorithm was developed and validated based on synovial fluid biomarkers, drawn at 24 h before surgery, in order to generate a preoperative PJI score. There were 104,090 samples used, with 10 synovial fluid biomarkers analyzed. The ML model achieved a diagnostic accuracy with 99.3% sensitivity and 99.5% specificity, validating alpha defensin, neutrophil percentage, and white blood cell count as the most important biomarkers [55].

## 4. Electronic Health Records and Risk Predictive Models for PJIs

Electronic health records (EHRs) contain a wide range of clinical information, including demographic data, comorbidities, medical history, and other health-related records. These EHRs contain massive structured and unstructured electronic data, referring to patient electronic data, that AI systems can use to improve diagnosis and prognosis. The natural language processing (NLP) based on DL models can be trained on these massive datasets, and can identify patterns that predict the risk of PJIs, helping clinicians gain insights into potential infectious risk assessment based on EHRs [56].

Given the high costs and economic burden that PJIs have, it is extremely important and equally efficient to identify high-risk patients, both pre-operatively and after a TJR surgery. With the aid of EHRs, a wide range of clinical data can be analyzed, including demographic data, past medical history, comorbidities, laboratory data, and others. Using ML models trained on such big datasets, patterns can be identified and harmonized with the occurrence of a PJI.

Predictive models allow clinicians to identify high-risk patients and modify and personalize the treatment based on these findings, to decrease the risk of developing a PJI. ML models used for assessment of a PJI risk can include logistic regression, random forest, support vector machines, and deep learning with neural networks. Table 3 presents a comparison of studies using machine learning for predicting periprosthetic joint infections using data from electronic health records.

In a recent retrospective study, Yeo et al. [57] have used five ML algorithms to predict superficial or deep surgical site infection following TKAs, on a total of 10,021 consecutive primary TKAs, at a minimum follow-up of 2 years. The supervised ML algorithms used were as follows: artificial neural network, stochastic gradient boosting, support vector machine, random forest, and elastic-net penalized logistic regression. Among these, the neural network model achieved the best performance in the conducted tests, with the strongest predictors of incidence for surgical site infections following primary TKAs being patient characteristics such as the Charlson comorbidity index, obesity, and smoking.

In another article comprising 1360 patients who underwent revision knee and hip arthroplasty procedures, the authors evaluated multiple classification models, including logistic regression, gradient-boosted classification trees, random forest, linear support vector machine, Gaussian support vector machine, and K-nearest neighbor, using clinical data extracted from the electronic health records. Among these models, the linear support vector model had the best performance in predicting PJIs [58].

Klemt et al. [59] retrospectively studied 618 revision TKA procedures and used an ML algorithm to assess outcomes. The ML model provided performance for predicting PJIs, with the strongest predictors of infection being irrigation and debridement regardless of modular component exchange, more than four prior surgeries, drug abuse, the presence of HIV infection or AIDS, metastatic disease, and obesity.

Wu et al. [60] developed a machine learning model based on data from electronic medical records to detect surgical site infections following total arthroplasties. In this study, 16,561 primary TKAs and 10,799 primary THAs were included, and nine XGBoost models were developed. Among these, the model that used both structured and unstructured (such as notes) data had the best performance.

## 5. Discussion

The reviewed studies clearly demonstrate the potential of AI in improving the early diagnosis of PJIs. Various models have been used depending on the type of clinical data—CNNs for radiographic imaging, support vector machines (SVMs) for MRI analysis, random forest models for biomarker-based predictions, and deep learning networks for processing electronic health records (EHRs). This reflects a growing trend in orthopedic research towards data-driven decision support tools [61].

Among imaging techniques, CNN-based models have shown particularly high accuracy in detecting early signs of infection, such as implant loosening, periosteal reactions, or joint effusions. However, many of these models have been tested only on small, institution-specific datasets, which raises concerns about their generalizability. Similarly, in MRI studies, while the application of SVMs has led to promising diagnostic performance, there is little information on external validation or reproducibility in other clinical settings.

In addition to imaging, AI techniques have also been applied to serum biomarkers and clinical data, as summarized in Table 4.

Table 4 summarizes AI applications in the analysis of biomarkers and electronic health records (EHRs) for PJI prediction. Logistic regression and random forest models applied to serum biomarkers identified CRP and fibrinogen as key indicators, but combining multiple markers did not improve diagnostic accuracy in some studies. A large-scale study using synovial fluid biomarkers achieved exceptional performance, with sensitivity and specificity over 99%, though its retrospective nature and single-lab data limit generalizability [53,55,56].

EHR-based models, particularly those incorporating free-text clinical notes using NLP, showed promise in risk prediction in real-world hospital settings. These models can access broad clinical information, but this advantage is shadowed by difficulties in data standardization and ethical use. Overall, both biomarker-based and EHR-based models may complement imaging approaches and offer value for earlier or preoperative risk stratification, especially when used in combination [57,58,59,60].

Biomarker-based models, especially those using random forests or ensemble learning, have identified combinations of inflammatory markers such as CRP, fibrinogen, or IL-6 as valuable predictors. Yet, the diagnostic advantage of combining multiple markers remains debated, and further prospective studies are needed [62]. Additionally, few models to date have focused on synovial fluid analysis, despite its established diagnostic value.

EHRs have also been explored using AI algorithms, particularly deep learning and natural language processing. These approaches are promising due to the large data volumes, but they also face challenges due to unstructured formats, incomplete documentation, and the need for data harmonization across systems. Moreover, ethical concerns such as patient privacy and data security should be considered when clinical implementation is desired [63].

Across all domains, a major limitation is the lack of standardized datasets and external validation. Most studies are retrospective and conducted in single centers, making it difficult to compare models directly or to establish benchmarks. Additionally, the use of different AI methods on heterogeneous datasets leads to variability in reported metrics, and hinders clear conclusions regarding which models are most effective in clinical practice [64].

Despite these limitations, the integration of AI into orthopedic infection diagnostics is a promising direction. Combining multiple data sources, including imaging, biomarkers, and clinical records, has the potential to improve early detection and guide personalized interventions. However, future research should prioritize multicenter studies, model interpretability, and clinical validation to bridge the gap between experimental performance and real-world utility [65].

These insights should guide future efforts to develop clinically validated, multi-modal AI systems for PJI prediction that are interpretable and applicable in routine orthopedic practice.

## 6. Challenges and Future Directions

Currently, AI use has been attempted in several Spanish hospitals, with the development and implementation of the AI-HPRO algorithm. This artificial intelligence model combines natural language processing (NLP) and extreme gradient boosting (XGBoost), and has been used for surgical site infection surveillance after hip replacement surgery. Results were promising, and after a validation cohort of 19,661 healthcare records, the AI-HPRO registered a high negative predictive value (99.98%), and reduced the number of clinical records reviewed in a manual manner by 88.95% [66].

In another paper, a meta-learner has been developed from the combination of five learning algorithms and has been used on a cohort of 323 patients with periprosthetic joint infections. The ML system was able to create adaptive diagnostic models and scored excellent results in comparison with the 2018 International Consensus Meeting (ICM) scoring system [67].

Combining multiple datasets, such as imagistic data, biomarkers analysis, and healthcare records, may provide an improved detection of early infection.

While AI and ML are gaining more attention, they have some limitations and disadvantages that need to be considered. ML algorithms have “the black box problems”, meaning that the process of choice is not transparent, and the final results are just “yes” or “no” with no other additional explanations [68].

ML offers promising results in terms of early and accurate detection of PJIs, but several challenges exist. ML algorithms require large and high-quality datasets in order to learn effectively. The next step, requiring validation in diverse patient populations, is important to guarantee robustness [69].

AI also presents ethical issues that need to be addressed, such as personal data privacy, bias, and informed consent. General Data Protection Regulation (GDPR) was first introduced by the European Union, and requires that all personal data be sufficiently protected [70]. This represents one of the most important concerns surrounding the use of AI and ML, and as in the development of an AI model relies on these data for training and validation, rigorous data security protocols must be implemented. To address these concerns, encryption techniques, access control, and strong authentication mechanisms are required to minimize the risks of personal data breaches [71]. As training should be performed on large datasets, mostly coming from EHRs, privacy data leaks may occur. To address this issue, anonymization and re-identification would be useful, though leaving some privacy risks unsettled [72]. Transparency and explainability are essential for gaining trust in healthcare, and the openness regarding the functioning and decision-making algorithms of AI is necessary. This could be addressed by providing clear and accessible documents for the development, validation, and performance of certain AI models [71].

Currently, researchers are developing CARE-AI (Collaborative Assessment for Responsible and Ethical AI implementation), a tool designed to advance the application of trustworthy and ethical AI prediction algorithms, to integrate the existing guidelines and identify possible gaps [73]. From the World Health Organization (WHO), a short guidance regarding Ethics and Governance of Artificial Intelligence for Health is available starting from 2021, which includes the six core principles that need to be protected, i.e., (1) protect autonomy, (2) promote human well-being, human safety, and the public interest, (3) ensure transparency, explainability, and intelligibility, (4) foster responsibility and accountability, (5) ensure inclusiveness and equity, and (6) promote AI that is responsive and sustainable [74].

## 7. Conclusions

Given the rise of consideration for AI and ML, research is still scarce, and gaps need to be filled. The importance of a rapid and early diagnosis of a PJI is crucial, as this complication may lead to poor outcomes. The ascent of AI technology in all domains provides a perspective for use in medical health prevention and early diagnosis, already demonstrating its potential in reviewing large amounts of data, translational research, and clinical and surgical analysis. Future development in PJI early diagnosis should emphasize multimodal data integration using AI and ML algorithms, especially combining serum biomarkers, imagistic data, pathology, and microbiology analysis.

While this review includes studies from various sources and methodologies, selection was based on relevance to PJI diagnosis, use of explicit AI or ML methods, and availability of performance metrics. Techniques varied significantly in terms of data type, algorithm complexity, and validation level. Deep learning models such as CNNs and ensemble methods have demonstrated high accuracy in image-based and synovial biomarker analysis, respectively, while support vector machines and random forests were more frequently applied to structured clinical data. Among these, only a few studies incorporated external validation or prospective design, highlighting the need for further clinical testing and real-world integration.

In summary, AI has demonstrated strong potential in enhancing early PJI diagnosis, particularly when models are applied to multimodal datasets. Future efforts should focus on clinically validated, explainable, and ethically sound AI tools to support orthopedic decision-making.

## Figures and Tables

**Table 1:** National burden of PJIs in the USA, Europe, and Korea, and the increase in total annual costs associated with PJIs.

Study	Region	Study Period	Total Annual Cost		
Premkumar et al. [5]	USA	2002–2017	USD 372,700,000	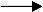	USD 902,900,000
Kim et al. [3]	Korea	2010–2018	USD 7,951,163	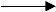	USD 17,982,388
Alt et al. [7]	Europe	2019	EUR 346,262,026		

**Table 2:** The use of different AI tools in imagistic diagnosis of PJIs: a comparative table summarizing AI applications across different imaging modalities.

Study Author	Imagistic Examination Used	Type of AI Used	Achieved Performance
Shah et al. [33]	X-rays	Convolutional neural network (CNN)	Accuracy: 88.3% Sensitivity: 70.2% Specificity: 95.6%
Li et al. [38]	CT (computer tomography)	Hierarchical GCN-Based Transformer (DL + multimodal)	Accuracy: 91.4% AUC: 95.9%
Galley et al. [39]	MRI (magnetic resonance imaging)	Compressed sensing-based slice encoding for metal artifact reduction 1.5 T	Sensitivity: 78–95% Specificity: 73–95%
Albano et al. [42]	MRI (magnetic resonance imaging)	Support vector machine (SVM)	Training set: Sensitivity 92% Specificity 62% Test set: Sensitivity 92% Specificity 79%
Nie et al. [47]	DBS (Dynamic Bone Scintigraphy)	Effective neural network (DBS-eNET), CNN	Periprosthetic knee infection: Accuracy: 87.74% AUC: 0.957 Periprosthetic hip infection: Accuracy: 86.36% AUC: 0.906

AUC = area under the curve.

**Table 3:** Comparison of studies using machine learning for predicting periprosthetic joint infections using data from electronic health records.

Study	Sample Size	ML Models Used	Best Performing Model	Data Type Used	Performance Metrics
Yeo et al. [57]	10,021 primary TKAs	ANN, SGB, SVM, RF, elastic-net LR	Artificial neural network (ANN)	Structured EHR data	AUC: ~0.79 (ANN)
Di Matteo et al. [58]	1360 revision TKAs and THAs	logistic regression, GBT, RF, linear and Gaussian SVM, KNN	Linear SVM	Structured EHR data	AUC: 0.81 (linear SVM)
Klemt et al. [59].	618 revision TKAs	ANN	ANN	Structured EHR data	AUC: 0.84
Wu et al. [60]	16,561 TKAs and 10,799 THAs	XGBoost models	XGBoost using structured + unstructured data	Both structured and unstructured EHR (incl. clinical notes)	AUC: 0.87 (best model using combined data)

TKAs = total knee arthroplasties; THAs = total hip arthroplasties; ML = machine learning; ANN = artificial neural network; SGB = stochastic gradient boosting; SVM = support vector machine; RF = random forest; elastic-net LR = elastic-net penalized logistic regression; GBT = gradient-boosted classification trees; KNN = K-nearest neighbor; EHR = electronic health record; AUC = area under the curve.

**Table 4:** Comparative analysis of AI applications for biomarker-based and EHR-based PJI detection.

Data Type	AI Method Used	Performance	Strengths	Limitations
Serum biomarkers	Logistic regression (LASSO) [53]	No gain over CRP/FIB alone	Inexpensive, widely available biomarkers	No diagnostic improvement from combining markers
Serum biomarkers	Random forest [56]	Not specified; key features: CRP, TP, BUN	Can identify most relevant lab predictors	No external validation; 133 cases
Synovial biomarkers	Ensemble ML model [55]	99.3% sensitivity/99.5% specificity	Extremely high performance; large dataset	Single-lab data; retrospective
EHR (structured + notes)	XGBoost + NLP [60]	Best model used both data types	Unlocks unstructured clinical information	Requires data harmonization; privacy concerns
EHR (structured)	SVM, RF, ANN, logistic regression [57,58,59]	SVM best in hip/knee PJI prediction	Useful for risk stratification, scalable	Often black box; mostly single-center studies

ANN = artificial neural networks; BUN = blood urea nitrogen; CRP = C-reactive protein; EHR = electronic health record; FIB = fibrinogen; LASSO = least absolute shrinkage and selection operator; ML = machine learning; NLP = natural language processing; RF = random forest; SVM = support vector machine; TP = total protein.

## Data Availability

The data presented in this study are available on reasonable request from the corresponding author.
